# miR-92a enhances recombinant protein productivity in CHO cells by increasing intracellular cholesterol levels

**DOI:** 10.1186/1753-6561-9-S9-P6

**Published:** 2015-12-14

**Authors:** Wan P Loh, Yuan S Yang, Andy Tan, Kong P Lam

**Affiliations:** 1 Bioprocessing Technology Institute, A*STAR, Singapore, 138668

## Background

MicroRNAs (miRNAs) have emerged as promising targets for engineering of CHO cell factories to enhance recombinant protein productivity. Manipulation of miRNA levels in CHO cells have been shown to improve product yield by their effects on cellular processes such as increasing proliferation, resisting apoptosis and enhancing specific productivity (qP). We previously demonstrated increases in qP and titer of CHO-IgG cells by over-expressing miR-92a [1]. Stably-transfected pools showed 27% increase in qP and 21% increase in titer compared to parental clone, without significant alteration in proliferation rates. The highest producing clone isolated from the miR-92a pool demonstrated 114% increase in qP and 88% increase in titer. However, the mechanisms by which miR-92a enhances qP in CHO cells are still uninvestigated.

## Materials and methods

To understand possible pathways via which miR-92a enhances productivity in CHO cells, we carried out transcriptomics study on 2 high-producing miR-92a clones (~33pg/cell-day) and blank-transfected pool (16pg/cell-day) using microarray. Genes identified to be differentially expressed in both clones relative to the blank-transfected pool were analyzed for pathway/gene ontology enrichment. Differential expression levels of Insig1 were confirmed using qPCR. Validation of insig1 as a direct target of miR-92a was done using a vector construct comprising of the 3' UTR sequence of insig1 downstream of luciferase reporter gene. Cholesterol assays were carried out on the cellular lipid extracts and the supernatant harvested from the miR-92a clones and blank-transfected pool. The clones were also stained with wheat germ agglutinin (WGA) conjugate for the visualization of the Golgi apparatus by confocal microscopy. The relative surface area of Golgi was calculated by normalizing surface area of Golgi compartment to that of the cell

## Results

Genes involved in cholesterol synthesis and metabolism were found to be enriched among the differentially expressed genes identified by microarray. Using qPCR, decreased expression levels of insig1 were found in transient pools and stable clones over-expressing miR-92a. Co-transfection of miR-92a mimic with the (luciferase-insig1 3'UTR) vector reduced luciferase expression by ~19% whereas miR-92a inhibitor significantly increased luciferase expression by 120%. INSIG1 inhibits cholesterol biosynthesis by preventing the activation of the SREBP transcription factor. The intracellular cholesterol concentration of both miR-92a clones were significantly increased by ~30% compared to the blank-transfected pool. The extracellular cholesterol concentrations for the miR-92a clones were also ~2-3-fold higher than that of the blank-transfected pool. Knock-out of miR-92a has been reported to reduce systemic cholesterol levels in mice [2], concurring with our observations that miR-92a over-expression increases cholesterol biosynthesis/secretion. Cholesterol is known to be essential for vesicular transport from the ER [3] and Golgi [4], and constitutes about 20% of the membrane lipid of the Golgi compartment. The relative surface area of Golgi was found to be 18-26% higher in the miR-92a clones compared to the blank-transfected pool (p<0.05).

## Conclusions

We have identified insig1 as a novel target of miR-92a. Our findings suggest that miR-92a may affect cholesterol metabolism by repressing insig1, resulting in raised intracellular cholesterol levels and increased Golgi volume and hence enhanced protein secretion.

## Acknowledgements

This study was supported by the Biomedical Research Council of Agency for Science, Technology and Research (A*STAR), Singapore.
